# Sex-specific signaling through Toll-Like Receptors 2 and 4 contributes to survival outcome of Coxsackievirus B3 infection in C57Bl/6 mice

**DOI:** 10.1186/2042-6410-3-25

**Published:** 2012-12-15

**Authors:** Brian J Roberts, Julie A Dragon, Mohamad Moussawi, Sally A Huber

**Affiliations:** 1 Department of Pathology, Center for Immunology and Infectious Disease, University of Vermont, Burlington, VT, USA; 2 Department of Microbiology and Molecular Genetics, University of Vermont, Burlington, VT, USA

## Abstract

**Background:**

Coxsackievirus B3 (CVB3) induces myocarditis, an inflammatory heart disease, which affects men more than women. Toll-like receptor (TLR) signaling has been shown to determine the severity of CVB3-induced myocarditis. No direct role for signaling through TLR2 had been shown in myocarditis although published studies show that cardiac myosin is an endogenous TLR2 ligand and stimulates pro-inflammatory cytokine expression by dendritic cells *in vitro*. The goal of this study is to determine which TLRs show differential expression in CVB3 infected mice corresponding to male susceptibility and female resistance in this disease.

**Methods:**

Male and female C57Bl/6 mice were infected with 10^2^ PFU CVB3 and killed on day 3 or 6 post infection. Hearts were evaluated for virus titer, myocardial inflammation, and TLR mRNA expression by PCR array and microarray analysis. Splenic lymphocytes only were evaluated by flow cytometry for the number of TLR+/CD3+, TLR+/CD4+, TLR+F4/80+ and TLR+/CD11c+ subpopulations and the mean fluorescence intensity to assess upregulation of TLR expression on these cells. Mice were additionally treated with PAM3CSK4 (TLR2 agonist) or ultrapure LPS (TLR4 agonist) on the same day as CVB3 infection or 3 days post infection to confirm their role in myocarditis susceptibility.

**Results:**

Despite equivalent viral titers, male C57Bl/6 mice develop more severe myocarditis than females by day 6 after infection. Microarray analysis shows a differential expression of TLR2 at day 3 with female mice having higher levels of TLR2 gene expression compared to males. Disease severity correlates to greater TLR4 protein expression on splenic lymphocytes in male mice 3 days after infection while resistance in females correlates to preferential TLR2 expression, especially in spleen lymphocytes. Treating male mice with PAM reduced mortality from 55% in control CVB3 infected animals to 10%. Treating female mice with LPS increased mortality from 0% in control infected animals to 60%.

**Conclusion:**

CVB3 infection causes an up-regulation of TLR2 in female and of TLR4 in male mice and this differential expression between the sexes contributes to disease resistance of females and susceptibility of males. While previous reports demonstrated a pathogenic role for TLR4 this is the first report that TLR2 is preferentially up-regulated in CVB3 infected female mice or that signaling through this TLR directly causes myocarditis resistance.

## Background

Myocarditis is a form of inflammatory heart disease which clinically affects men (2:1) over women [1]. In our model of virus-induced myocarditis, coxsackievirus, a small positive sense ssRNA picornavirus causes an autoimmune reaction in the heart following infection. Autoimmunity most likely results from antigenic mimicry between viral and heart antigens [2]. Despite similar levels of viral replication, male mice develop myocarditis whereas females do not. Several mechanisms have been reported for the sex bias associated with myocarditis including estrogen, γδ T-cells, CD1d, Regulatory T-cells and more recently, Toll-Like Receptor (TLR) expression [3-8]. TLRs have been implicated in several autoimmune disease, including systemic lupus erythematosus, type 1 diabetes, autoimmune encephalomyelitis, and autoimmune myocarditis [8-14]. TLRs are a family of proteins which play a key role in innate immune defense. Unlike the adaptive immune response which is highly specific to a distinct antigen and takes up to 10 days from microbe exposure to optimally develop, signaling through TLRs is immediate. TLRs recognize common motifs uniquely shared by classes of different pathogens called pathogen associated molecular patterns, or PAMPs. Currently, 11 TLRs have been identified in mammals, each interacting with a specific PAMP including lipids, proteins and nucleic acids [15]. TLRs can show sex bias in expression; such bias has been shown for TLR7 and TLR9. TLR7 and TLR9-mediated functions are promoted on pDCs by signaling through estrogen receptor α [16,17]. In contrast, estradiol may suppress increased expression of TLR4 after LPS stimulation [18,19]. No information on sex bias of other TLRs, including TLR2, is available.

The goal of this communication was to evaluate whether the sex bias in CVB3 induced myocarditis susceptibility was mediated by differences in TLR expression between male and female mice. PCR array and microarray analysis were conducted on CVB3 infected male and female mice at days 0, 3, and 6 post infection. Female mice were found to have higher levels of cardiac TLR2 mRNA at 3 days post infection (p.i.) compared to males. Male mice have increased levels of TLR4 protein on splenic lymphocyte populations compared to females. Treatment of male mice with PAM3CSK4, synthetic triacylated, lipopeptide (a TLR2-specific ligand), at the time of infection abrogates the mortality normally associated with coxsackievirus infection, whereas female mice treated with ultrapure LPS, a TLR4 specific ligand, at day 3 post infection resulted in much greater mortality than observed in female mice treated with virus and PBS alone. These results indicate that TLR2, expressed in female mice during the early infection period confers a protective effect, whereas TLR4 expressed at higher levels in male mice is lethal.

## Methods

### Mice

Male and female C57Bl/6 mice were purchased from the Jackson Laboratories, Bar Harbor Maine. Mice were housed at the University of Vermont in sterile ventilator cages. Adult mice ages 6-8 weeks were used in all experiments. Experiments consisted of groups starting with a minimum of 5 mice and were repeated at least two times. Differences in final mouse numbers between sexes and treatments were due to mortality. All experiments were reviewed and approved by the University of Vermont Institutional Animal Care and Use Committee.

### Virus

The H3 variant of CVB3 was derived from an infectious cDNA clone which has been described previously [20]. Mice were infected by intra-peritoneal (i.p.) injection of 0.5ml of phosphate-buffered saline (PBS) containing 10^2^ plaque forming units (PFU) of the virus.

### Organ viral titers

Hearts were aseptically removed, perfused with PBS, and weighed before being homogenized in RPMI-1640 media (Mediatech, Manassas, VA) containing 2% fetal bovine serum, antibiotic/mycotic, penicillin and streptomycin. Cellular debris was removed by centrifugation at 300xg for 10 minutes and the supernatants were subjected to a series of 10-fold serial dilutions in RPMI-1640-2%FBS and titers were determined by plaque-forming assay on HeLa cell monolayers as described previously [20].

### Toll-Like receptor agonists

Both the TLR2 ligand Pam3CSK4, a synthetic triacylated, lipopeptide and the TLR4 ligand Ultrapure LPS isolated from *E.coli 0111.B4* were purchased from Invivogen San Diego, CA. Both ligands were resuspended in endotoxin free water and diluted in PBS for i.p. injection. PAM3CSK4 was injected at a concentration of 50 ug/mouse [21,22], and UP-LPS was injected at a concentration of 20 mg/kg [23,24].

### Lymphocyte preparation

Spleen were aseptically removed and processed through a fine-mesh screen to produce single-cell suspensions. Lymphocyte suspensions were centrifuged over Histopaque (Sigma Chemical Co., St. Louis, MO).

### Mouse TLR pathway PCR array

Male and female C57Bl/6 mice were infected and harvested on day 0 (uninfected), 3, or 6 post infection. Hearts were perfused with 2 ul/ml ribolock RNase inhibitor (Fermentas, Maryland, USA) and incubated 2- 4 days in RNAlater (Qiagen, California, USA) according to manufacturer’s directions. Following perfusion with ribolock, 1/3 of the heart was removed and prepared for histology as described. The remaining heart tissue was cut to 10 mg and homogenized in trizol (Sigma-Aldrich, Missouri, USA) with a biospec mini- bead beater (Cole-Parmer, Illinois, USA). RNA was extracted with chloroform using the Qiagen RNeasy Mini RNA isolation Kit (Qiagen, California, USA.) Prepared RNA samples were evaluated for quality and quantity at the Vermont Cancer Center’s Microarray facility. Three representative hearts from each group were chosen based first on histology score to ensure infection, then based on RNA quality and amount of RNA recovered. An aliquot of each samples were pooled by sex and day and run with the S.A. Bioscience RT2 Profiler PCR Array Mouse TLR Pathway PCR Array (PAMM-018) (SA Bioscience, Qiagen-USA, Valencia CA) at the Vermont Cancer Centers Microarray Facility at the University of Vermont.

### Microarray

RNA samples used in the PCR Array were further subjected to microarray analysis. Three representative hearts from each group were chosen based first on histology score to ensure infection, then based on RNA quality and amount of RNA recovered. Samples were individually run on the Affymetrix Mouse Gene 1.0st Array Chip. Individual results were averaged by group and submitted to the University of Vermont Bioinformatics group for analysis.

### Calculation of probe set statistics and differential expression

RMA expression statistics from the 12 samples were modeled in a 2 × 3 block design, sex by day 0, 3, and 6 post infection, with mouse modeled as random effect. Pairwise linear modeling was conducted using ANOVA as implemented in Partek® Genomics Suite™, version 6.6 (Copyright^©^ 2009, Partek Inc., St. Louis, MO, USA). ANOVA provided the response (fold change calculated using the least square mean) and the p-value associated with each probe set, as well as a step-up, adjusted p-value for the purpose of controlling the false discovery rate.

A second ANOVA was performed on the target genes chosen from the results of the super array, thus improving the statistical power to detect enrichment in those probe sets.

Microarray data has been submitted to the Gene Expression Omnibus, and we are currently awaiting their reply.

### RTqPCR

Samples for RTqPCR validation were prepared as described for the microarray. RNA samples validated by RTqPCR were independent of those used in the PCR Array and microarray. Samples were analyzed for TLR2 expression with the Applied Biosystems TaqMan® Gene Expression Assay for mouse TLR2 (kit# Mm01213946_m1) (Applied Biosystems, Carlsbad, CA.) at the Vermont Cancer Center’s DNA facility at the University of Vermont.

### Antibodies

FITC conjugated anti-CD3 (clone 17A2), APC-Cy7 or PerCp-Cy5.5 conjugated anti-CD4 (clone RMA-5), APC conjugated anti-CD11c (clone HL3), APC-Cy7 conjugated anti-CD8a (clone 53-6.7), Alexa 647 conjugated anti-IL4 (clone 11B11), and PE conjugated anti-IFNγ (clone XMG1.2) were purchased from BD Pharmagin, San Diego, CA. PerCp-Cy5.5 conjugated anti-F4/80 (clone BM8), Alexa 647 or PE conjugated anti-TLR2 (clone 6C2), and PE conjugated anti-TLR4 (clone UT41) were purchased from eBioscience, San Diego, CA. Antibodies were diluted 1:100 in PBS containing 1% Bovine Serum Albumen (BSA). Negative controls were anti-rat IgG2a conjugated with the same fluorochromes used with the antigen-specific antibodies. All antibody mixtures contained 1:100 rat anti-mouse CD16/CD32 (Fc Block; clone 2.4G2).

### Flow cytometry

#### Surface marker staining

1 × 10^5^ isolated lymphocytes were washed in PBS-containing 1%BSA and resuspended in 0.1ml PBS-1%BSA containing 1:100 dilution flourochrome conjugated antibodies and 1:100 dilution of Fc Block (anti-CD16/CD32). Cells were stained in the dark at 4°C for 15 minutes, washed twice with 1XPBS-1%BSA and fixed with 1XPBS containing 2% parafolmaldehyde for flow analysis. Cells were analyzed using a BD LSR II flow cytometer using a single excitation wavelength (488 nm) and band filters for PerCp-Cy5.5 (695/40nm), FITC (525 nm), PE (575 nm) and APC-Cy7 (633 nm). The excitation wavelength for Alexa 647 is 643 nm and a band filter of 660/20 nm. The cell population was classified for cell size (forward scatter) and complexity (side scatter). A minimum of 10,000 cells were evaluated. Positive staining was determined based on isotype controls.

#### Intracellular cytokine staining

1 × 10^5^ spleen cells were cultured for 4 hours in RPMI-1640 medium containing 10% FBS, antibiotics, 10 ug brefeldin A (BFA: Sigma), 50 ng/ml phorbol 12-myristate 13-acetate PMA: Sigma) and 500 ng/ml ionomycin (Sigma). The cells were washed in PBS-1% bovine serum albumin (BSA: Sigma) containing BFA (BS-BSA-BFA), incubated on ice in PBS-BSA-BFA containing 1:100 dilution of FC Block, anti-CD4, and anti-CD8a. Cells were washed with PBS-BSA-BFA, fixed for 10 minutes in 2% parafolmaldehyde (PFA) and resuspended in PBS-BSA containing 0.5% saponin containing 1:100 dilutions Fc Block, Normal Rat Serum, anti-IL4, and anti-IFNγ for 15 minutes on ice. Cells were washed with PBS-BSA-saponin followed PBS-BSA and resuspended in 2% PFA.

#### Histology

Hearts were fixed in 10% formalin, sectioned and stained with hemotoxylin and eosin. Sections were blindly evaluated by an experienced member of the laboratory on a scale of 0 to 4 where 0 represents no inflammation, 1 represents 1 to 10 lesions per section, 2 represents 11-20 lesions per sections, 3 represents 21 to 40 lesions per section, and 4 represents greater than 40 lesions per section. Mice with a score of 0 in the pancreas were assumed to be uninfected and removed from data analysis.

### Statistical analysis

**Figure 1:**
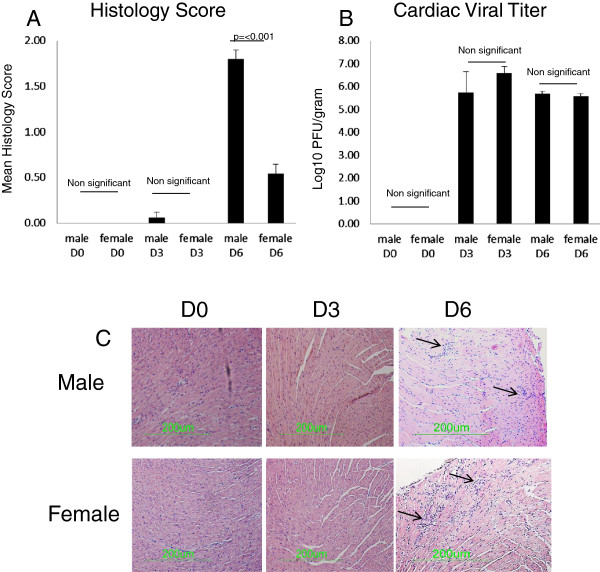
**Male mice develop more severe myocarditis than female mice despite equivalent viral titers.** Figure 1A. Male mice have higher levels of inflammation than female mice at the Day 6 timepoint (p=<0.001). Histology was scored blindly on a 1-4 scale by an experienced member of the lab. Figure 1C show representative micrographs, note that the black arrows point to areas of inflammation. Figure 1B shows cardiac viral titers as log10 PFU/gram. Despite differences in histology score, no significant difference was observed in viral load between male and female mice at either Day 0, 3 or 6 post infection. Numbers of mice used in this experiment were as follows: Males D6=7, D3=7; Female D6=14, D3=9).

Students T-test was used to determine differences between individual mice for histology, organ viral titers and flow cytometry using SPSS PASW Statistics 18. Statistics for the agonist histology and titer date (Figure 1) were analyzed by oneway analysis of variance to compare sexes by treatment groups. A priori pairwise contrasts comparing each treatment within sex and comparing similar treatments between sexes were run. Mortality was measured by the Mantel-Cox Log rank test using GraphPad Prism 5. Flow cytometry graphs are presented as mean number of cells positive spleenocytes for a specific marker or as mean fluorescent intensity (MFI) of the specified TLR. Error bars are given as the standard error of the mean (SEM).

## Results

### Male C57Bl/6 mice are more susceptible to CVB3 myocarditis than females

Male and female C57Bl/6 mice were infected with 10^2^ PFU CVB3 and evaluated for myocarditis (Figures 1A and 1B) and cardiac virus titers (Figure 1C) at 3 and 6 days post infection. Control mice were uninfected. Myocarditic inflammation was not observed in either male or female mice 3 days post infection, but by day 6, both male and female mice showed signs of cardiac inflammation with male mice having a higher myocarditis score than female mice (mean cardiac score 0.54±0.11 for females and 1.75±0.11 for males, p<0.001). Despite increased myocarditis in males, there was no significant difference in cardiac virus titer at either day 3 or 6 between the sexes (Figure 1C).

**Figure 2:**
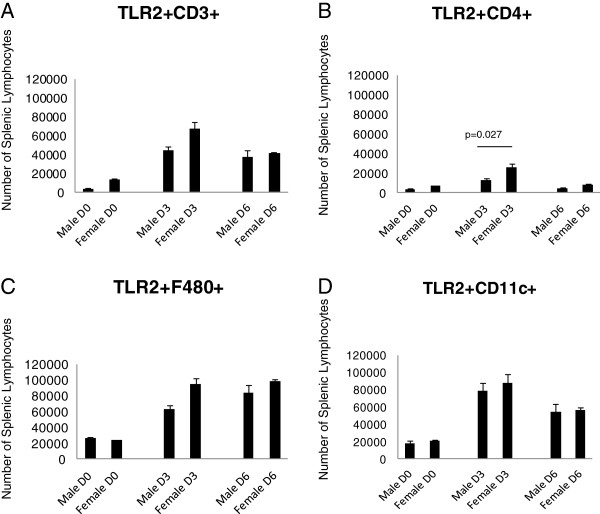
**Surface marker staining by flow cytometry demonstrates that at the Day 6 timepoint, female mice have greater numbers of TLR2-expressing T-cells (CD3) (p=0.036), macrophages (F4/80) (p=0.006) and dendritic cells (CD11c) (p=0.019) compared to males.** Numbers of mice used in this experiment were as follows: Male D6=6, D3=3, D0=3; Female D6=5, D3=4, D0=5.

### Super and microarray expression

A targeted expression study using the S.A. Bioscience RT2 Profiler PCR Array Mouse TLR Pathway superarray was conducted on cardiac RNA isolated from infected male and female mice harvested on day 0, 3, and 6 post infection. The results of this assay suggested TLR2 was differentially expressed in males and females at day 3 post infection, with females having greater expression of TLR2 compared to males; data not shown. The primary goal of the microarray assay was to repeat validate the super array results with a prospective hypothesis of gender specific differential expression during the course of infection on a genome-wide level and with independent replicates. With this prospective hypothesis, TLR2 was found to be significantly differentially expressed in the interaction between gender and day of infection from pre-infection to 3-days post, and from 3-days post to 6-days post (Table 1). Data obtained from both the superarray and the microarray was further validated by RT-PCR conducted on infected cardiac samples independent of those used in the arrays. Table 2 shows the relative fold change for TLR2 based on the three independent RNA assays.

**Table 1:** A table of expression statistics for the gender by day-post-infection interactions for the incremental time points

**A.**			
Interaction between gender and 3-day post-infection, ie (F*D3 – F*D0) – (M*D3 – M*D0)			
Target Gene	FDR	Fold Change	P-Value
TLR2	1.58e-2	1.27	1.58e-2
**B.**			
Interaction between gender and 6-day versus 3-day post-infection, ie (F*D6 – F*D3) – (M*D6 – M*D3)			
Target Gene	FDR	Fold Change	P-Value
TLR2	2.92e-3	−1.37	2.92e-3

These represent the difference of differences for the expression across the two factors.

**Table 2:** The relative fold change for TLR2 based on the three mRNA assays for the interaction between sex and 3-day post-infection, and gender and 6-day versus 3-day post-infection

**Target Gene: TLR2**	**Fold Change, PCR Array**	**Fold Change, micorarray**	**Fold Change, RTPCR (ave. ΔCT)**
Gender by 3-day post infection	9.624	1.27	1.495693
Gender by 6-day vs. 3-day post infection	−12.923	−1.37	0.995534

### Lymphocyte subpopulations show sex-specific differences in TLR expression

**Figure 3:**
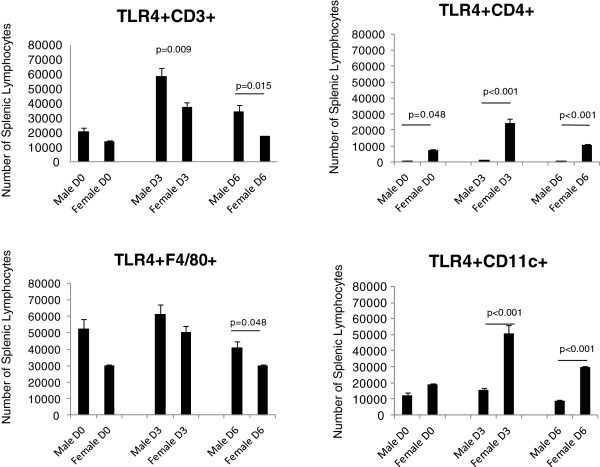
**Male mice show an increase in the number TLR4 expressing CD3+ cells (p=0.009) and macrophages (p=0.048) at the Day 6 timepoint.** In contrast, female mice have greater numbers of TLR4 expressing CD4+ cells at all three timepoints (p=<0.001, 0.016, and <0.001 respectively), and dendritic cells at Days 3 (p=0.019) and Day 6 (p=<0.001). Numbers of mice used in this experiment were as follows: Male D6=6, D3=3, D0=3; Female D6=5, D3=4, D0=5.

**Figure 4:**
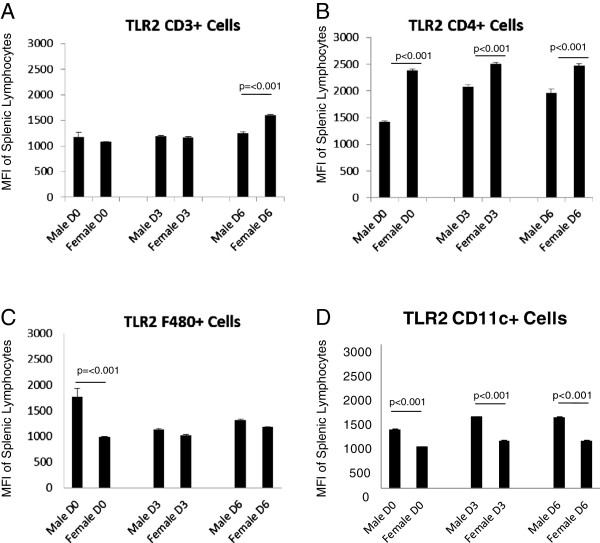
**Mean Fluorescent Intensity (MFI) data shows sex differences in TLR2 protein expression on lymphocyte populations.** Female mice have higher levels of TLR2 expression on CD3+ cells at Day 6 (p=<0.001) compared to male mice. In addition, CD4+ cells isolated from female mice have higher levels of TLR2 expression at all three timepoints compared to males (p=0.001, 0.001, and 0.006 respectively). In contrast, male mice have higher levels of TLR2 expression on macrophage populations at Day 3 (p=0.006) and Day 6 (p=<0.001) relative to female mice. Male mice also show greater expression of TLR2 on dendritic cells at all three timepoints (p=0.002, <<0.001, and 0.006 respectively). Numbers of mice used in this experiment were as follows: Male D6=6, D3=3, D0=3; Female D6=5, D3=4, D0=5.

**Figure 5:**
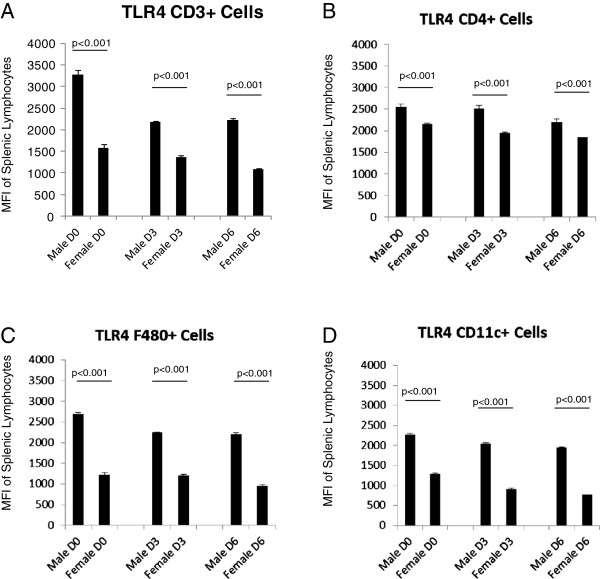
**MFI shows sex differences in TLR4 protein expression on lymphocyte populations.** Male mice have greater levels of TLR4 expression on CD3+, F480+ and CD11c+ cells at all three timepoints (p=<0.001 for all values). In addition, male CD4+ cells have higher levels of TLR4 at both Day 3 (p=0.039) and Day 6 (p=0.011) compared to females. Numbers of mice used in this experiment were as follows: Male D6=6, D3=3, D0=3; Female D6=5, D3=4, D0=5.

To determine if the observed differences in TLR expression also occurred in lymphoid cells at the protein level, spleens of the male and female mice were removed and processed for analysis by flow cytometry. While microarray analysis showed sex differences in TLR2 expression, a role for TLR4 in CVB3 myocarditis has also been shown [25-27]. We were curious to see if there was a sex bias in TLR4 expression on lymphoid cells, and included analysis of this TLR in these experiments. Analysis of TLR expression in male and female spleen cells based on individual cell types [CD3+, CD4+, F4/80+ (monocytes) or CD11c+ (dendritic cells)] is shown for both number of cells/ spleen (Figures 2 and 3) and the mean fluorescence intensity (MFI) which describes the relative amount of TLR expressed per positive cell (Figures 4 and 5). Evaluation of TLR4 expression was more complex (Figure 4). TLR4+CD4+ cells were increased in all uninfected and infected female mice compared to equivalent male animals. Infected female mice had increased numbers of TLR4+CD11c+ cells compared to male mice, however no changed was observed in uninfected mice. Male mice harvested at day 6 have increased numbers of TLR4-expressing CD3+ (p=0.009) and F480+ cells (p=0.048). MFI data showed greater expression of TLR2 on female CD4+ cells at all three timepoints (p=0.001, 0.001, 0.0036 respectively) and on CD3+ cells at day 6 (p=<0.001). Male mice, on the other hand, had increased expression of TLR2 on F480+ cells at days 3 and 6 (p=0.006 and <0.001 respectively) as well as on CD11c+ cells at all three days (p=0.002, <0.001, and 0.006 respectively) (Figure 4). Expression levels of TLR4 tended to be increased in infected males compared to female lymphocytes at both day 3 and 6 post infection (Figure 5). These results indicate that there are inherent differences in TLR expression in both the heart and in lymphoid cells in mice early after infection (day 3) prior to inflammation in the heart.

**Figure 6:**
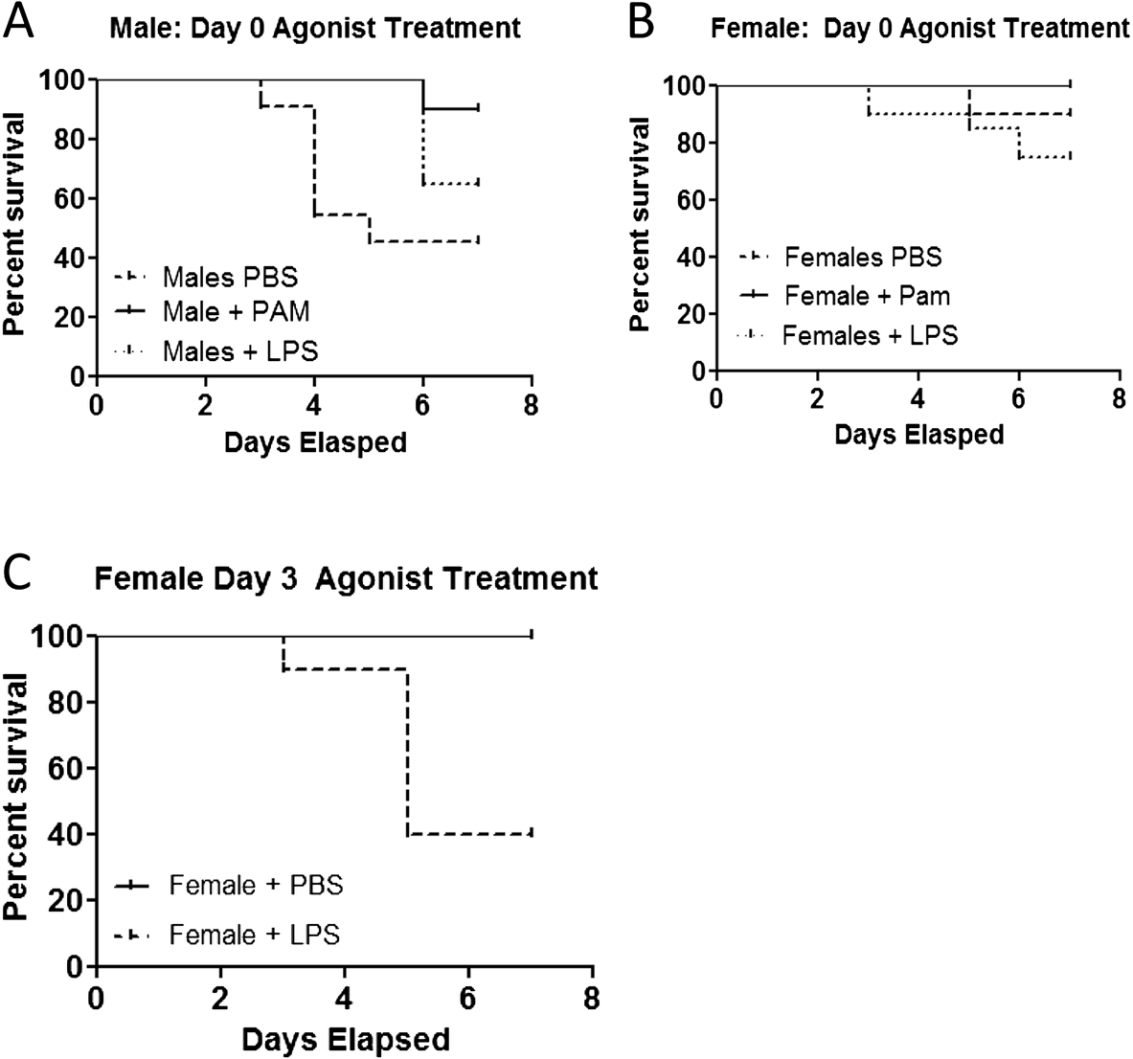
**Treatment of infected mice with TLR 2 or 4 agonist effects survival.** Treatment of male mice with PAM3CSK4 at the time of infection results in better survival (p=0.0261) than those given PBS or LPS which have no difference in survival (Figure 6A). Treatment of female mice with either agonist at the time of infection does not alter survival (Figure 6B). Administration of LPS to female mice on D3 p.i. results in 60% mortality compared to 0% mortality seen with PBS controls (p=0.0046) (Figure 6C). Male mice treated with LPS at Day 3 had 100% mortality (data not shown). Numbers of mice used in these experiments were as follows: Male PBS=10, PAM=9, LPS=16; Female PBS=9, PAM=5, LPS=17.

### Treatment with TLR 2 and 4 agonists alters sex differences in disease mortality

Further evidence for the role of TLR2 and TLR4 in CVB3 myocarditis was obtained by treating male and female C57Bl/6 mice with either 50μg PAM3CSK4 (PAM; TLR2 agonist) or 20 mg/kg Ultra-Pure LPS (UP-LPS; TLR4 agonist). Control mice were infected and treated with vehicle control (PBS). Infected males treated with PBS showed 55% mortality by day 7 post infection compared to only 10% mortality in similarly infected females (Figure 6). Treatment of male mice with PAM along with infection significantly reduced mortality to 10% compared to control mice (p=0.0261). Treatment of males with LPS delayed mortality but did not significantly reduce total animal deaths by day 7 post infection. Treating infected females with either PAM or LPS had minimal effect on mortality compared to infected PBS treated animals. There was no significant difference in mortality in female mice treated with PAM or LPS compared to PBS controls. Treating female mice with LPS on D3 p.i. resulted in 60% mortality compared to 0% mortality seen with PBS controls (p=0.0046) indicating that early stimulation of TLR2 confers a protective effect whereas TLR4 stimulation causes mortality.

**Figure 7:**
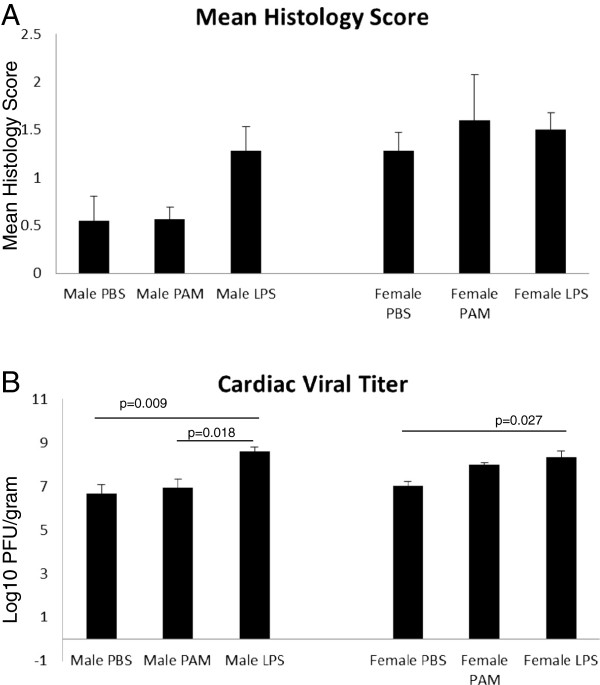
**TLR agonist treatment effects viral titer but not histology score. **Male and female mice treated with either LPS or PAM show no difference in mean histology score (Figure 7A). Figure 7B shows that male mice treated with LPS have greater viral replication in the heart compared to those treated with either PBS (p=0.005) or PAM (p=0.002). Treatment of female mice either PAM (p=0.001) or LPS (p=0.001) results in greater viral replication compared to those treated with PBS. In addition, female mice treated with PAM have greater levels of viral replication compared to male mice given the same treatment (p=0.021). Numbers of mice used in these experiments were as follows: Male PBS=10, PAM=9, LPS=16; Female PBS=9, PAM=5, LPS=17.

Infected female mice treated with PAM show an increase in cardiac viral titer compared to PBS controls (p=0.001), however no effect was seen in cardiac inflammation (Figure 7). Male mice treated with PAMs showed no difference in cardiac inflammation or viral titer compared to PBS controls. Treatment of male mice with LPS resulted in both increased viral titer (p=0.026) and inflammation (p=0.005) compared to PBS controls. LPS treatment of male mice additionally resulted in an increase in viral titer (p=0.002) and histology score (p=0.032) compared to those treated with PAM. Similar to the results seen with PAM treatment, female mice treated with LPS have increased viral titers (p=0.001) compared to PBS controls, however there was no observed difference in myocarditis score.

## Discussion

This communication shows that there is a significant difference in TLR2 and TLR4 expression between CVB3 infected male and female mice at both the mRNA in the heart and protein level in lymphoid cells. It should be noted however that while the initial observations of sex differences in TLR expression were made based on PCR array and microarray analysis of infected cardiac tissue, we have also noted the existence of these differences on splenic lymphoid populations by flow cytometry. Further, it shows that signaling through TLR2 and TLR4 has basically different effects on CVB3 pathogenicity with TLR2 signaling providing partial protection and TLR4 signaling providing increased pathogenicity, at least in males. The observation of TLR4 aggravation of myocarditis in males is not unexpected as studies by Fairweather and colleagues have previously shown that TLR4 expression is significantly increased in CVB3 infected male BALB/c mice and that blocking TLR4 reduces myocarditis [27]. Other studies have shown that TLRs-3, -7, -8, or-9 modulate enteroviral myocarditis [14,28-37]. However, these studies do not concentrate on potential sex differences in TLR expression or role in myocarditis susceptibility. Nor has the role of TLR2 in CVB3 myocarditis been adequately investigated. A recent study has shown that cardiac myosin acts as an endogenous ligand for TLR2 and 8 and stimulates dendritic cells in vitro to release pro-inflammatory cytokines [38]. Since myocyte lysis is induced by either virus replication or host immune response to the virus, infection should release cardiac myosin into the local environment, causing one to anticipate that TLR2 could have a major impact on pathogenicity. However, it was surprising that TLR2 signaling actually induced more protection that aggravated pathogenicity. Based on the in vitro evidence of enhanced pro-inflammatory cytokine response, one would have expected TLR2 signaling to promote pathogenicity. The reason for the difference between the in vitro activation of dendritic cells and the protection observed in whole mice subsequent to CVB3 infection may be reflected in the complex effects of CVB3 infection on TLR2 and TLR4 up-regulation in different cell populations. The number of TLR2+ cells is greatest in female mice at day 6 compared to males and can be seen most prominently on CD3+, F480+ and CD11c+ cells. Interestingly, the amount of TLR2 expression on cells isolated from female mice is greatest on CD3+ and CD4+ cells at days 3 and 6. Male mice, however, have greater expression of TLR2 on macrophages and dendritic cells at both days 3 and 6. Imanishi *et al* showed that direct TLR2 signaling of T cells stimulates production of IFNγ [39], a cytokine previously shown to be essential for autoimmunity in this model of CVB3 induced myocarditis [40]. If TLR2 expression is increased on dendritic cells in male mice, in vitro activation using cardiac myosin might induce pro-inflammatory cell responses from cells. However if TLR2 expression on T cells from male mice is suppressed, there may be less direct activation of T cell populations. This might be important as TLR2 signaling in T cells has been shown to promote Tregulatory cell responses [41,42]. Thus, increased TLR2 expression on T cells in females may explain the increased Tregulatory cell response observed in CVB3 infected female mice [43,44].

Why sex differences occur in TLR expression is not completely understood. Certain of the TLR genes, such as TLR-8 and TLR7 are on the sex chromosomes or their expression is controlled by the sex chromosomes [45,46]. While most genes on the X chromosome undergo x-inactivation in females to prevent dose response differences between males and females, some genes can escape inactivation. Also, TLR7 has been shown to translocate to the Y chromosome which would also affect its expression [46]. TLR2 and TLR4 are not on the sex chromosomes, however but on chromosome 3 and chromosome 4, respectively in the mouse [47]. Cytokines can modulate TLR expression on immune cells [48], and it is well established that sex hormones alter cytokine responses with estradiol and testosterone having distinct effects on pro- and anti-inflammatory cytokines [49]. Therefore, it is reasonable that the TLR expression profiles may vary between the sexes.

In contrast to TLR2 enhancement of T-regulatory cell activation, signaling through TLR4 may have the opposite effect. Frisancho-Kiss *et al* reported that T cell Ig mucin 3 (TIM3) decreases cardiac inflammation caused by CD11b+ cells while at the same time increasing CD4+/CD25+/FoxP3+ Treg populations [50]. Further studies from their laboratory have shown that male mice have increased levels of TLR4 expression on macrophages found in the heart following infection. Expression of TLR4 is thought to increase the production of IL-18 which increases IFNγ production through the MyD88 signaling pathway and is likely responsible for Th1 polarization seen in male mice [8,51]. Our data shows that male mice at all three time points have higher levels of TLR 4 expression on T-cells and macrophages. Since antigen presenting cells such as DCs and macrophages are responsible for providing the cytokine environment to polarize T-cells it makes sense that APCs of male mice would have higher levels of TLR4 expression which in turn would result in greater levels of IL-18 production leading to the production of more IFNg and Th1 cells [52].

The finding of increased viral replication in animals treated with the TLR2 agonist was unexpected. However, it is known that specific cytokines/chemokines can alter coxsackievirus replication. Most notable of these are the type 1 interferons [53] and CXCL10 [54]. These cytokines/chemokines may either directly affect virus replication or alter virus load in the target tissue through their activation of innate effectors such as natural killer cells. There is an inverse correlation between the ability of cardiotropic viruses to induce Type 1 interferons and their ability to cause myocarditis [55]. Since TLR activation is the classical method for inducing interferon response, it is reasonable that these molecules might affect virus load, and TLR2 activation has been found to be crucial to control of other viruses [56]. Therefore, the question remains why TLR2 activation should enhance virus load in the heart in CVB3 infection when it has been shown to promote virus clearance in other viral models. One possibility is the nature of the virus receptors. Decay accelerating factor (DAF) is one of the two known cellular receptors for coxsackievirus B3 [57]. Microbial infections can up-regulate expression of DAF [58] which in the case of CVB3 may lead to enhanced virus replication. This would be especially true if TLR2 engagement simultaneously promoted Tregulatory cell activation which suppressed anti-viral host responses. A similar explanation for why signaling through TLR2 and TLR4 enhances viral load comes from the shared adaptor protein MyD88. Studes by Fuse et al have shown that MyD88-/- mice have reduced viral load and develop less myocarditis by day 7 than intact controls. These results suggest that MyD88 is important in the development of the pathology accociated with infection. In addition, levels of the coxsackievirus-adenovirus receptor (CAR-one of the two known receptors for CVB3 which crucial for its internalization) are decreased in the cardiac tissue of MyD88-/- mice compared to control animals. Finally, their study shows that IRF-3 is increased in the hearts of MyD88-/- mice. IRF-3 expression results in increased type I IFN production which is responsible for antiviral effects. [59]. While it has been shown that a deficiency in TLR4 resulted in higher viral titers it is important to note two key differences in our methods. First, the TLR4-/- mice used were on the Balb/c backrgound, whereas our mice were C57Bl/6. Secondly they evaluated cardiac viral titers at days 2 and 12 post infection, whereas our TLR agonist-treated mice were harvested on day 7 [26]. Of similar interest, female PBS-treated mice had slightly higher myocarditis when compared to male mice treated with PBS. While this is highly unusual given that male mice have been repeatedly shown to be more susceptible, there is a possible explanation for this observation. It has been shown that low doses of estrogen actually promote a Th1 cytokine response (IFNγ) whereas high doses of estrogen favor are Th2 IL-10 response [60,61]. We have previously shown that susceptibility of female mice changes during the different phases of the ovarian cycle with mice infected during the proesterus phase being more susceptible than those infected during the estrus or metestrus phases. It is therefore likely that these mice could have had the appropriate hormone environment to allow for greater susceptibility [62]. We feel, however, that what is most important to take away from the agonist studies are the differences that the two agonists have on myocarditis within the sexes in terms of their effect on disease susceptibility.

## Competing interests

The authors declare that they have no competing interests.

## Authors’ contributions

BR provided scientific design, data analysis and management and completed the majority of the laboratory bench work (infection, agonist treatment RNA preparation, flow cytometry and viral titers) and wrote the manuscript. JD provided assistance in RT-qPCR, microarray and PCR array experimental design and provided statistical analysis and figures for data based on gene array experiments. MM participated in organ tissue harvesting and animal husbandry. SH provided scientific design and manuscript review. All authors read and approved the final manuscript.
